# Retraction: Protein Kinase C beta Mediates CD40 Ligand-Induced Adhesion of Monocytes to Endothelial Cells

**DOI:** 10.1371/journal.pone.0295636

**Published:** 2023-12-05

**Authors:** The PLOS ONE Editors

Following the publication of this article [1], concerns were raised regarding Figs 1, 2, and 6 Specifically:

- In Fig 1A there appears to be an overlap between the 20ng/mL CD40L and 40ng/mL CD40L panels. Additionally, there are several cells in the 40ng/mL CD40L panel that do not appear in the 20ng/mL CD40L panel despite the overall overlap.
- The actin panel in Fig 2B appears similar to the actin panel in Fig 6A despite representing different experimental conditions.

The authors did not respond to the concerns raised and the underlying data was not provided for editorial review. The *PLOS ONE* Editors remain concerned about the issues in Figs 1, 2, and 6 which cannot be resolved in the absence of the original underlying data.

In light of the concerns affecting multiple figure panels that question the integrity and reliability of these data, the *PLOS ONE* Editors retract this article.

All authors either did not respond directly or could not be reached.
